# Epigenetic Gene Mutations Impact on Outcome in Acute Myeloid Leukaemia

**DOI:** 10.1016/j.ebiom.2015.04.020

**Published:** 2015-05-22

**Authors:** Christine S. Young, Kathryn M. Clarke, Ken I. Mills

**Affiliations:** Blood Cancer Research Group, Centre for Cancer Research and Cell Biology, Queen's University Belfast, Belfast, Northern Ireland, UK

Acute myeloid leukaemia (AML) is a heterogeneous clonal disorder arising in the myeloid lineage with an average age of around 62 years at diagnosis. Morphological and cytogenetic analysis has identified a number of sub-types with a wide range of survival outcomes. Some of the more favourable outcomes are associated with patients who harbour balanced reciprocal chromosomal translocations including the t(15;17) translocation resulting in a fusion gene between PML and RARα; this group of patients have acute promyelocytic leukaemia (APL) (Freireich et al., 2014). Over the past few years, next-generation sequencing has assisted in the identification of a spectrum of molecular mutations in many of the other sub-types of AML, particularly in those with an apparent normal karyotype.

Clinical trials have shown that patients with t(15;17) respond very well to All-Trans-Retinoic-Acid (ATRA) or to ATRA with arsenic trioxide (ATO) resulting in some excellent outcomes in which there is an 85–90% 5-year overall survival (Coombs et al., 2015). ATRA has been widely used in the treatment of APL due to its ability to specifically bind to the ligand-binding domain of the RARα portion of the fusion protein, resulting in the terminal differentiation and subsequent apoptosis of the leukaemic promyelocytes. ATO which targets the PML portion has been combined with conventional ATRA therapy resulting in degradation of the fusion protein. Furthermore, with this therapeutic combination, chemotherapy could be omitted for the patients who have low risk APL (Coombs et al., 2015).

Unfortunately, even with the high rates of remission and overall survival, there remains a sub-set of APL patients who do not respond to ATRA/ATO and it is these patients who highlight the need for better stratification upon diagnosis. An appropriate sub-division of patients’ needs to be applied at diagnosis, patients who would not respond to, for example ATRA/ATO, could be treated with the most suitable therapy based on their clinical and molecular presentation.

The study published in this edition by Shen et al. (2015) has highlighted the need to stratify patients as they demonstrate a more heterogeneous molecular picture associated with APL than previously considered.

APL has been shown to require only the PML–RARα fusion protein, however Shen et al. (2015) in their study of 535 APL patients in two cohorts (training; n = 266 and testing; n = 269) showed by systematic analysis of genetic markers the presence of a range of additional mutations typically associated with normal karyotype AML patients in patients with APL. The most common mutations were FLT3-ITD or -TKD (15.8%), WT1 (4.7%) and N-RAS (4.5%); although the FLT3 mutation rate was lower than reported (43%) in a previous study within the UK (Gale et al., 2005). However, if epigenetic modifier genes (EMGs) such as DNMT3A, TET2, IDH1, IDH2 and ASXL1 were considered as a group, then 6.5% of APL patients had EMG mutations. Overall, almost 1/3 of patients (30.6%) had a least one mutation and the EMGs were often associated with other mutations.

Furthermore, when the APL patients were stratified using Sanz's risk scores (Sanz et al., 2000), over half (50.4%) of the high-risk patients were more likely to harbour more than 2 mutations in addition to PML–RARα. Of these, those with EMG mutations were associated with a poorer outcome. Patients in the lower risk groups tended to have a less complex mutational burden: 23.1% in low risk and 25.0% in the intermediate groups. A similar landscape of mutated genes was seen in each of the risk groups.

Shen et al. (2015) also showed a connection between mutational burden and response to ATRA/ATO therapy: patients in the low-risk groups responded to treatment better than those in the intermediate and high-risk groups. However, the biggest discriminator for both overall survival and disease free survival, in the testing and training data sets, was not FLT3 mutations but was the presence of mutations in the EMG. This would point towards screening patients at diagnosis and the development of a stratification model encompassing the presence of EMG mutations as a predictive indicator of resistance to treatment with ATRA/ATO.

This study further confirms that ATRA/ATO therapy is a highly effective treatment for APL but clearly highlights the rationale for alternative approaches targeting non-responding patients. Shen et al. (2015) identified subsets of mutated genes contributing to a previously unnoticed group of APL patients with poorer outcome and provides the opportunity for the development of better targeted therapies to treat high-risk disease. The inclusion of other mutations outside of PML–RARα has opened up the possibility that APL should be regarded as a more complex heterogeneous disease and ultimately may contribute to the improvement of the current stratification regimen currently employed in the clinic.

Future studies, in larger cohorts and in different centres, should address the need to validate the use of other agents in combination with ATRA/ATO such as epigenetic modifying agents. This has been alluded to in preclinical studies demonstrating the potential capacity for combination with HDAC inhibitors (De et al., 2014). However, a novel stratification model needs to be developed, at the point of diagnosis, to identify those APL patients who are resilient to ATRA/ATO therapy.

## Authors' Contributions

CY, KC and KIM all contributed to the data interpretation and writing aspects of the manuscript. CY and KC should be considered as making an equal and joint contribution as 1st authors.

## Conflicts of Interest

The authors declare that they have no conflict of interests in writing this commentary.
